# Soluble Sugars and Sucrose-Metabolizing Enzymes Related to Cold Acclimation of Sweet Cherry Cultivars Grafted on Different Rootstocks

**DOI:** 10.1100/2012/979682

**Published:** 2012-05-02

**Authors:** Ece Turhan, Sergul Ergin

**Affiliations:** ^1^Department of Horticulture, Faculty of Agriculture, Eskisehir Osmangazi University, 26160 Eskisehir, Turkey; ^2^Department of Horticulture, Faculty of Agriculture, Uludag University, 16059 Bursa, Turkey

## Abstract

The bark tissues were collected from 4-year-old sweet cherry trees cvs. 0900 Ziraat and Lambert grafted on Gisela 5 and Mazzard rootstocks in cold-acclimated (CA) and nonacclimated (NA) stages. Bark tissues subjected to 4°C and −5°C injured to a limited extent in both stages. However, more than 50% injury occurred by temperatures equal to or colder than −15°C only in NA period. Total soluble sugar (TSS), reducing sugars, and sucrose contents were higher in CA than those in NA stages in all samples. The activities of acid invertase (EC 3.2.1.26) and sucrose synthase (SS) (EC 2.4.2.13) enzymes were higher in NA stage than those in CA stage. Considering the rootstocks, reducing sugars were higher in both cultivars grafted on Gisela 5 whereas sucrose contents were higher in both cultivars grafted on Mazzard. However, the enzyme activities of both cultivars were higher on Mazzard rootstock than on Gisela 5. In conclusion, cold hardiness of sweet cherry graft combinations was suggested by increasing their TSS, reducing sugars, and sucrose contents significantly in the CA stage. Moreover, acid invertase and SS are down regulated during cold acclimation. Indeed the results suggested that Mazzard is more cold-hardy rootstock than Gisela 5.

## 1. Introduction

Cold and frost are important environmental factors that limit geographic distribution of plants and crop yields worldwide [1, 2]. Many plants from temperate and cold climates, including many important crop species, increase in freezing tolerance when exposed to low temperatures. This process on increasing tolerance is known as cold acclimation or cold hardening [3, 4].

Studying cold hardiness of woody plants is complicated, because freezing injury occurring in the field usually only becomes visible in spring when growth commences. A range of different methods can be used to evaluate injury after artificial freezing in controlled conditions [5]. The most frequently used methods for freezing injury assessment are visual rating of injury and electrical conductivity (EC) of diffused electrolytes [6].

Plant cells undergo dehydration during freezing stress due to the presence of ice in extracellular spaces [3]. Membrane damage is mainly due to the dehydration that occurs during the freeze-thaw cycle. Freezing-induced destabilization of the plasma membrane involves different types of lesions [7, 8].

It has been reported that cold acclimation is accompanied by biochemical changes including the expression of cold-stress proteins, such as dehydrins [9], the accumulation of sugars, particularly sucrose [10], sugar alcohols (mannitol and inositol) [11], the accumulation of other cryoprotectants, such as glycinebetaine [12] and proline [13], changes in lipid composition [8, 14], in sugar metabolizing enzymes [15–19], and enhancements of antioxidative mechanisms [9].

The accumulation of sucrose, other simple sugars, and osmolytes that typically occurs with cold acclimation also seems to contribute to the stabilization of membranes [9] and may play a key role in protecting the proteins from freezing and dehydration [7]. Physiologically, compatible solutes should have no adverse metabolic effects even at very high concentrations. They are thought to stabilize sensitive cellular components under stress conditions and also act as bulk osmoprotectants [2].

The enzymes of carbohydrate metabolism are essential for growth, development, and carbohydrate partitioning in sink organs. Invertase, sucrose phosphate synthase (SPS), and SS are directly involved in sucrose synthesis and/or degradation [19]. The invertases (B-D-fructofuranosidase, soluble acid, neutral and cell wall-bound acid) catalyze the irreversible hydrolysis of sucrose to glucose and fructose [20]. Invertases normally reside within the cell wall or vacuole and provide higher osmoticum to cold-acclimated cells [21]. SS (UDP-D-Glc: D-Fru 2-a-glucosyltransferase, EC 2.4.2.13) is a cytoplasmic enzyme that catalyzes the reversible cleavage of sucrose with uridine 5′-diphosphate (UDP) to form UDP-glucose and fructose. Although capable of synthesizing sucrose, SS functions primarily in the direction of sucrose degradation [22]. The alterations of these enzymes have been investigated in wheat [15, 18], in spinach [16], and in cabbage [19] exposed to low temperature.

Turkey ranks first in the world in sweet cherry production with ~400.000 t [23]. Cold hardiness of sweet cherry cultivars and rootstocks is important in sweet cherry cultivation. Mahaleb rootstocks are usually considered hardier than Mazzard [24]. On the other hand sweet cherry cultivars grafted on the rootstock Colt are less hardy than on either Mazzard or Mazzard × Mahaleb. Moreover sweet cherry cultivars grafted on Mazzard × Mahaleb rootstock were hardier than those grafted on Mazzard or Mahaleb rootstocks [25].

Emphasis is on sweet cherry, because this is currently one of the most important fruit-tree crop, and frost damage risk, is poorly understood. Therefore, it is important to elucidate the mechanism and environmental factors that affect freezing tolerance. This will help to prevent frost injury and achieve stable crop production. There is, however, no information available on sugar metabolism and the enzymes involved in sucrose metabolism in sweet cherry during cold acclimation. The objective of this study was to examine seasonal changes in sugar and sucrose-metabolizing enzymes and their relationship between cold hardiness in sweet cherry graft combinations.

## 2. Materials and Methods

### 2.1. Plant Material

One-year-old shoots of sweet cherry tree cv. 0900 Ziraat and Lambert grafted on Gisela 5 and Mazzard rootstocks were collected from 4-year-old trees in Bayramic, Turkey in CA (in January) and NA (in July) stages. In January, the average temperature was 4.6°C (range −8.95°C–16.73°C). In July, the average temperature was 25.8°C (range 11.28°C–37.57°C). Ten shoots were collected randomly from each 3 or 4 trees of each graft combinations and packed on ice in 3 replicates and brought to the laboratory. The shoots were separated into two groups for the analysis. One part of the shoots was processed for controlled freezing test to measure electrolyte leakage. Other part of the shoots was used in sugars and enzyme analysis.

### 2.2. Low Temperature Treatments

Samples were exposed to low temperatures according to the method of Arora et al. [26]. Middle part of the collected shoot pieces, 20–25 cm long, were wrapped in aluminum foil along with moistened paper and placed in manually controlled low-temperature freezer. Plant tissue temperature was monitored with a copper-constant thermocouple (Testo 925, Omni Inst., Scotland, UK) inserted in the foil pouch. Temperature was decreased stepwise, as approximately 1.5°C/h to −5°C and 5°C/h thereafter. Samples were exposed to low temperature at 4, −5, −15, and −25°C for 12 h. Samples were then removed from the freezer at each temperature and placed at 4°C overnight for slow thawing. In the next step, bark samples were scraped off using a razor blade. The bark samples were used to determine electrolyte leakage.

### 2.3. Determination of Freezing Injury

Freezing injury of bark tissues at each temperature was determined by measuring electrolyte leakage as described previously by Eris et al. [27]. Briefly, bark tissues with 1 × 1 cm dimensions were cut from the shoots. They were lightly rinsed in distilled water, gently blotted with paper towel, and placed in test tubes (one bark piece per test tube). Ten mL of distilled water was added to test tubes which were then vacuum infiltrated to allow uniform diffusion of electrolytes. Tubes were shaken on a gyratory shaker (250 rpm) for 4 h at room temperature. Electrical conductivity of each sample was measured using WTW TetraCon 325 conductivity meter (InoLab Cond Level 1, Weilheim, Germany). Electrical conductivity of each sample was measured once more after the tubes were autoclaved (0.12 MPa, 120°C, 20 min) and cooled. Percentage injury at each temperature was calculated from ion leakage data using the equation [26]: % injury = [(%L_(t)_ − %L_(c)_)/(100 − %L_(c)_)] × 100, where %L_(t)_ and %L_(c)_ are percentage ion leakage data for the treatments and control samples, respectively. All measurements were replicated three times.

### 2.4. Soluble Sugars

Sugars were extracted by suspending 100 mg of barks in 5 mL of 80% (v/v) ethanol in an 85°C water bath for 1 h and then collecting the ethanolic liquid. This procedure was repeated four times for 1 h, 30 min, 15 min, and 15 min. The ethanolic solutions were combined and evaporated to dryness at 55°C with the aid of continuous ventilation. The dried sugars were dissolved in 1 mL of distilled water and kept frozen at −20°C until determination.

TSS and sucrose concentrations were determined by the anthrone reagent method, as modified for the determination of nonreducing sugars [28] by a Beckman UV-DU 520 spectrophotometer (Beckman Coulter, Fullerton, CA, USA) at 620 nm using glucose and sucrose as the standards, respectively. Reducing sugar concentrations were determined colorimetrically with dinitrosalicylic acid [29] using glucose as the standard at 550 nm.

### 2.5. Sucrose-Metabolizing Enzymes

Soluble (cytosolic) acid invertase activity in bark tissue was determined according to Aloni et al. [30]. In short, tissue samples of approximately 500 mg were ground in 5 mL ice-cold grinding medium containing 25 mM HEPES buffer (N2-2-ethanesulphonic acid) pH 7.2, 5 mM MgCl_2_, 2 mM DDT (DL-Dithiothreitol) and 3 mM DIECA (diethyldithiocarbamic acid) as antioxidant. This mixture was centrifuged at 20 000 g for 20 min at 4°C. Aliquots of 100 *μ*L of the supernatant were incubated in 10 mL 0.1 N phosphate citrate buffer pH 5.0 and 20 mM sucrose. The incubation was carried out for 30 min at 37°C and was terminated by addition of 1 mL dinitrosalicylic acid reagent. After boiling for 5 min, the resulting sugars were determined colorimetrically. SS activity was determined according to Aloni et al. [31]. Following extraction as described for acid invertase the mixture was dialysed overnight in order to remove the internal sugars. The enzymatic activity was determined as sucrose breakdown on aliquots of 200 *μ*L incubated in incubation medium containing 0.1 M phosphate-citrate buffer pH 7.0, 200 mM sucrose, and 5 mM UDP. After incubation at 37°C for 30 min, the resulting fructose was determined by the dinitrosalicylic acid reaction. The data were expressed on fresh mass basis. Total soluble protein contents of the crude enzyme extracts were determined according to Bradford [32].

### 2.6. Statistical Analysis

The experiment was arranged in a randomized block design with three replications. Data were tested by SPSS 13.0 for Windows program and mean separation was accomplished by Duncan test at *P* < 0.05.

## 3. Results

### 3.1. Freezing Injury

**Figure 1:**
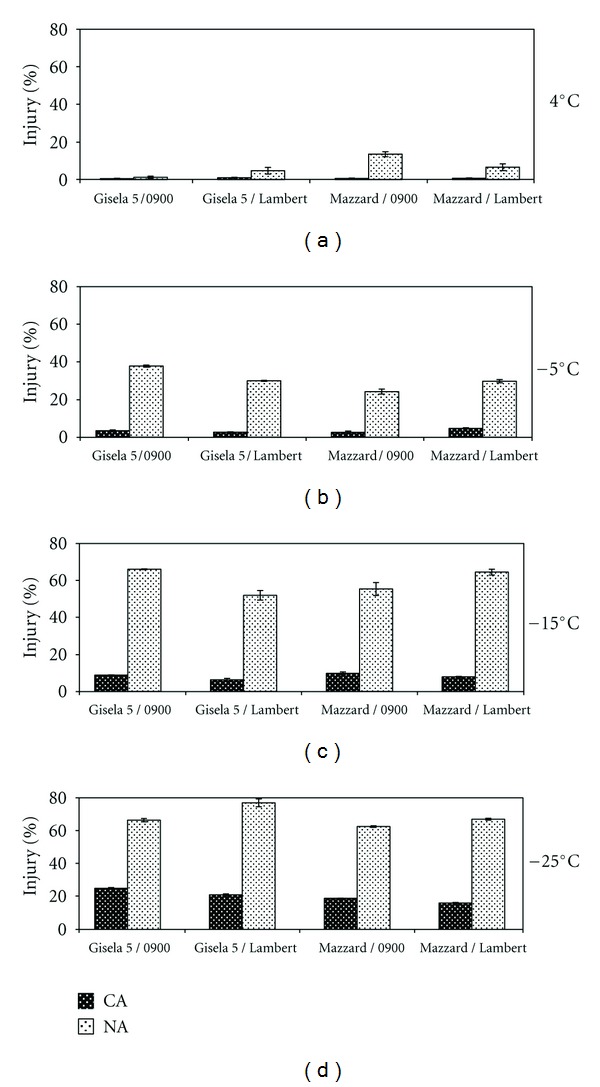
The changes of injury under low-temperature treatments in cold-acclimated (CA, in January) and nonacclimated (NA, in July) bark tissues of sweet cherry cultivars grafted on different rootstock. Error bars represent ± SE of three replications.

Changes in freezing injury in bark tissues of sweet cherry tree cv. 0900 Ziraat and Lambert grafted on Gisela 5 and Mazzard rootstocks in cold-acclimated (CA, in January) and nonacclimated (NA, in July) stages with respect to exposure to freezing treatments are shown in Figure 1. In general, freezing injury (expressed by reference to controls) was the highest in NA stage than CA stage after freezing tests. The lowest and the highest average freezing injury were observed in barks exposed to 4°C and −25°C, respectively. Freezing injury was below 50% in barks exposed to 4°C and −5°C in both period and was higher in NA stage exposed to −15°C and 25°C.

Difference between graft combinations was more prominent in NA stage than in CA stage. Accordingly, while there was no significant correlation between graft combinations and low temperature treatments at 4, −5, and −15°C, injury was significantly greater in sweet cherry cultivars grafted on Gisela 5 rootstock compared with Mazzard at −25°C treatment. The highest injury by this treatment was observed in cv. Lambert grafted on Gisela 5. However, Lambert constituently showed higher injury on Mazzard than 0900.

### 3.2. Soluble Sugars

**Figure 2:**
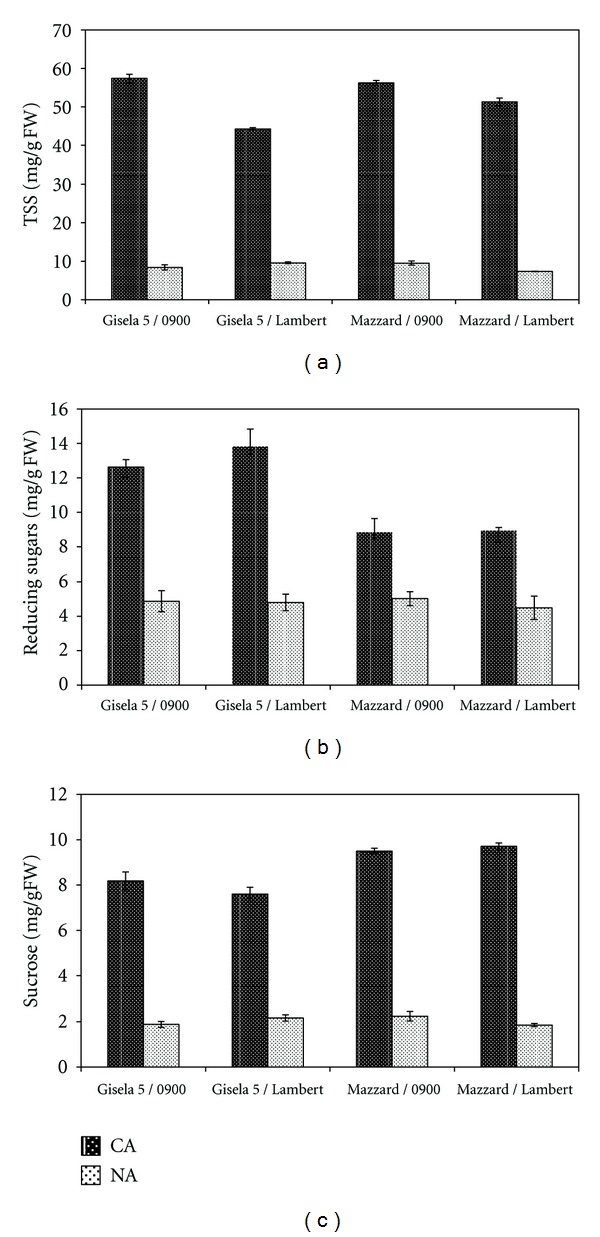
TSS (a), reducing sugars (b), and sucrose (c) contents in cold-acclimated (CA, in January) and nonacclimated (NA, in July) bark tissues of sweet cherry cultivars grafted on different rootstock. FW: fresh weight. Error bars represent ± SE of three replications.

TSS contents of all graft combinations were significantly higher in CA stage than NA stage (Figure 2(a)). The highest TSS content was measured in Gisela 5/0900 (~57.5 mg/g FW) and Mazzard/0900 (~56.5 mg/g FW) combinations and the lowest in Gisela 5/Lambert (~44.5 mg/g FW). However, no significant difference was detected among TSS contents of graft combinations in NA stages. Two-way ANOVA revealed a significant effect of sampling stage, grafting combination, and the interaction of sampling stage and grafting combination on TSS content (Table 1).

Reducing sugar content was significantly greater in CA stage than in NA stage in all graft combinations (Figure 2(b)). Reducing sugar content was higher in either sweet cherry cultivars grafted on Gisela 5 rootstock compared with Mazzard rootstock in CA stages. The highest and the lowest reducing sugar contents in CA stage were detected in Gisela 5/Lambert (~14.0 mg/g FW) and Mazzard/0900 (~9.0 mg/g FW) combinations, respectively. Similar to TSS content in NA stages, no significant difference was detected in reducing sugar content of the graft combinations in NA stages. Two-way ANOVA revealed a significant effect of sampling stage, grafting combination, and the interaction of sampling stage and grafting combination on reducing sugar content (Table 1).

All graft combinations had significantly higher sucrose contents when sampled at CA than at NA stage (Figure 2(c)). When data from CA period were considered, the highest sucrose content was observed in Mazzard/Lambert combination (~9.7 mg/g FW) and the lowest Gisela 5/Lambert (~7.6 mg/g FW). As with TSS and reducing sugars, there were no significant differences among graft combinations in sucrose content in NA period. Two-way ANOVA revealed a significant effect of sampling stage, grafting combination, and the interaction of sampling stage and grafting combination on sucrose content (Table 1).

### 3.3. Sucrose-Metabolizing Enzymes

**Figure 3:**
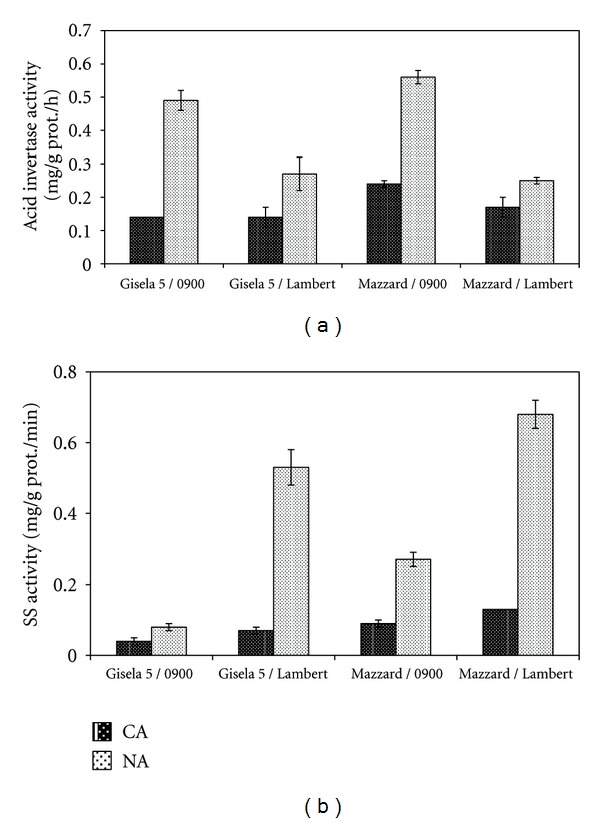
Acid invertase (a) and SS (b) activities in cold-acclimated (CA, in January) and nonacclimated (NA, in July) bark tissues of sweet cherry cultivars grafted on different rootstock. Error bars represent ± SE of three replications.

Acid invertase activity of all graft combinations indicated significant differences between CA and NA stages (Figure 3(a)). Acid invertase enzyme activity was significantly greater in NA stage than in CA stage in all graft combinations. The highest and the lowest enzyme activity in CA stage were detected in Mazzard/0900 and in either sweet cherry cultivars grafted on Gisela 5 rootstocks, respectively. The highest acid invertase activity in NA stage was detected in Mazzard/0900 (0.56 mg/g prot./h) while the lowest activity was detected in Mazzard/Lambert combination (0.25 mg/g prot./h). Two-way ANOVA revealed a significant effect of sampling stage, grafting combination, and the interaction of sampling stage and grafting combination on acid invertase activity (Table 1).

SS activity of all graft combinations was significantly higher in NA stage compared with those in CA stage (Figure 3(b)). In addition, SS activity varied among graft combinations in both CA and NA stages. When data from the CA period were considered, the highest SS activity was measured in Mazzard/Lambert combination (0.13 mg/g prot./min) and the lowest in Gisela 5/0900 (0.04 mg/g prot./min). The order of the graft combinations with the highest and the lowest SS activity in NA stage was similar to that in CA stage. Two-way ANOVA revealed a significant effect of sampling stage, grafting combination, and the interaction of sampling stage and grafting combination on SS activity (Table 1).

## 4. Discussion

### 4.1. Freezing Injury

Cell membrane stability was widely used to express stress tolerance; and higher membrane stability could be correlated with abiotic stress tolerance [33]. In order to determine the response of sweet cherry cultivars grafted different rootstocks to low temperatures (4°C, −5°C, −15°C, −25°C), we measured membrane thermostability by electrolyte leakage method. Regarding the results of injury from bark tissues, it was the highest in NA stage than CA stage in all grafting combinations (Figure 1). Beside that, in the NA stage, higher than 50% injury was determined in bark tissues exposed to −15°C and −25°C treatments in all graft combinations. The injury was significantly greater in sweet cherry cultivars grafted on Gisela 5 rootstock compared with that on Mazzard at −25°C. The greatest injury by −25°C treatment was observed in cv. Lambert grafted on Gisela 5 with ~77%. These results are in a good agreement with the result of other studies which shows that low temperature had usually been considered as the major cause of increased cell membrane permeability, relative conductivity, and injury index of plant tissue [26, 27, 34, 35]. To our knowledge, this is the first detailed study of membrane stability in sweet cherry cultivars/rootstock carried out under laboratory freezing tests.

### 4.2. Soluble Sugars

It is well known that sugar metabolism is affected by temperature stress, and sugars accumulate in response to low-temperature stress [36]. Our results indicate that during NA period, bark tissues had significantly lower TSS contents than during the CA period (Figure 2(a)), which paralleled their freezing tolerance. Increases in TSS ameliorate the impact of dehydration associated with freezing [9]. Consequently, seasonal changes in TSS content were related to changes in cold hardiness and air temperatures in sweet cherry. Accumulation of TSS during cold acclimation occurs in several species, such as peach [37], raspberry [36], olive [27, 38], cabbage [19, 39], arabidopsis [40], and wheat [41].

In this study, reducing sugar and sucrose content were significantly greater in CA stage than in NA stage in all graft combinations (Figures 2(b) and 2(c)). However, reducing sugar content was higher in either sweet cherry cultivars grafted on Gisela 5 rootstock compared with Mazzard rootstock in CA stages (Figure 2(b)). On the other hand, sucrose content was higher in either sweet cherry cultivars grafted on Mazzard rootstock compared with Gisela 5 rootstock in CA stages (Figure 2(c)). The most commonly accumulated soluble sugar in response to low temperature is sucrose [16]. However, sugar accumulation at low temperature is not limited to only sucrose. The types of sugar accumulated vary among plant species during cold acclimation. It was determined that the contents of sucrose, glucose, and fructose increased on exposure to low temperature in spinach [16] and in cabbage [39]. Hamman et al. [42] found that the high ratio of glucose plus fructose to sucrose are positively correlated with hardiness in grape. Palonen [36] also reported that high concentrations of soluble carbohydrates, sucrose, and a high ratio of sucrose to glucose plus fructose were characteristic of a hardy raspberry cultivar. Similarly, it was reported that changes in TSS, particularly glucose and sucrose contents were related to variations in freezing tolerance of olive [27, 38].

However in the present study reducing sugar but not sucrose was most abundant sugar in bark tissues of 1-year-old shoots. Beside that, the proportional increase in sucrose content in CA and NA stages was greater than that of reducing sugars (Figures 2(b) and 2(c)). These results are in good agreement with those of Gulen et al. [38], who detected that reducing sugar, but not sucrose, was the major soluble carbohydrate and the proportional increase in sucrose content in CA and NA stages was greater than that of reducing sugars in olive leaves.

### 4.3. Sucrose-Metabolizing Enzymes

Invertase, SPS, and SS are directly involved in sucrose synthesis and/or degradation [19]. However, changes in activities of these enzymes at low temperature varied between the plant species. In the present study, acid invertase and SS activity were significantly greater in NA stage than in CA stage in all graft combinations (Figures 3(a) and 3(b)). In reality, there have been different reports about this subject in the literature. Castonguay and Nadeau [17] reported that, when acid invertase and SS activities in alfalfa decreased during fall acclimation, SPS and galactinol synthase (GS) showed markedly higher activity at low temperature. Similarly, Guy et al. [16] found that leaf SPS activity was significantly increased by the low-temperature treatment, whereas SS and invertases were not in spinach. On the other hand, Calderon and Pontis [15] showed that the activity of SS rose continuously, immediately after the chilling shock in wheat. Beside that, both enzymes activities increase by cold treatments in wheat [18]. Sasaki et al. [19] suggest that SS and SPS, but not acid invertase, are regulated by cold acclimation and deacclimation and play important roles in sugar accumulation and acquisition of freezing tolerance in the leaves of cabbage seedlings.

In conclusion, the grafting combinations in sweet cherry investigated here increased their cold hardiness by increasing their TSS, reducing sugars, and sucrose contents significantly in the CA stage. On the other hand, acid invertase and SS are downregulated during cold acclimation. To better understand the regulation of cold-induced accumulation of soluble sugars, it should be measured the activity of other key regulatory enzymes involved in the metabolism of carbohydrate.

## Figures and Tables

**Table 1:** Results of variance analysis (ANOVA) of stage (S), grafting combination (GC), and their interactions with total sugar content (TSS), reducing sugars content, sucrose content, acid invertase activity, and sucrose synthase activity (SS) in bark tissues of sweet cherry cultivars grafted on different rootstock. Numbers represent *F* values at 0.05 level.

Dependent variable	Independent variable		
	S	GC	SxGC
TSS content	11743.831*	56.068*	67.153*
Reducing sugars content	229.261*	9.661*	9.084*
Sucrose content	2410353*	13.811*	15.107*
Acid invertase activity	138.761*	24.881*	13.735*
SS activity	358.408*	82.003*	53.250*

*Significant at *P* < 0.05.

## Abbreviations

CA: Cold acclimated
EC: Electrical conductivity
GS: Galactinol synthase
NA: Nonacclimated
SPS: Sucrose phosphate synthase
SS: Sucrose synthase
TSS: Total soluble sugar.
